# Comparative analysis of biologic drug effects and distal particulate embolization among three drug-coated balloons: a histological and biological study in a rabbit model

**DOI:** 10.1007/s12928-026-01290-2

**Published:** 2026-06-09

**Authors:** Kazuki Aihara, Sho Torii, Manabu Shiozaki, Yu Sato, Kaho Hashimoto, Daiki Suzuki, Ryosuke Ohmura, Yuto Ono, Yuki Matsumoto, Norihito Nakamura, Yuji Ikari, Gaku Nakazawa

**Affiliations:** 1 Department of Cardiology, Tokai University School of Medicine, 143 Shimokasuya, Isehara, 259-1193 Kanagawa Japan; 2 Faculty of Medicine, Department of Cardiology, Kindai University, Sakai, Japan

**Keywords:** Peripheral artery disease, Endovascular treatment, Chronic limb-threatening ischemia, Drug-coated balloons, Drug-eluting stents

## Abstract

Drug-coated balloons (DCBs) have improved clinical outcomes in femoropopliteal interventions; however, distal particulate embolization remains a critical concern, particularly in patients with chronic limb-threatening ischemia. This study aimed to histologically and biologically compare local drug effects and distal embolization among three contemporary DCBs: Ranger, IN.PACT Admiral, and Luminor. Eighteen healthy rabbits underwent descending aorta balloon angioplasty followed by DCB inflation. Animals were divided into three groups (*n* = 6 each): Ranger (2.0 µg/mm², 3-minute inflation), IN.PACT (3.5 µg/mm², 3-minute inflation), and Luminor (3.0 µg/mm², 1-minute inflation). At 28 days, treated aortas and downstream skeletal muscles were evaluated histopathologically and for tissue paclitaxel concentrations. In the target lesions, median paclitaxel concentrations were comparable among the Ranger, IN.PACT, and Luminor groups (3047.5, 1537.1, and 551.5 fmol/mg, respectively). Consistent with this, histological evaluations demonstrated robust and comparable medial smooth muscle cell loss across all devices. Conversely, evaluation of downstream skeletal muscles revealed striking differences in distal drug washout. Distal muscle paclitaxel concentrations were significantly higher in the Luminor group (1069.4 fmol/mg) compared to the IN.PACT (354.8 fmol/mg) and Ranger (173.8 fmol/mg) groups. Histologically, the incidence of distal particulate embolization was most severe in the Luminor group (median 4.0), followed by the IN.PACT group (3.0), both higher than the Ranger group (1.0). The low-dose Ranger DCB achieved robust drug delivery with significantly lower distal embolization risk than high-dose DCBs. These mechanistic insights may guide device selection, but clinical studies are needed to confirm limb outcomes.

**Graphical abstract:**

**Figure:**
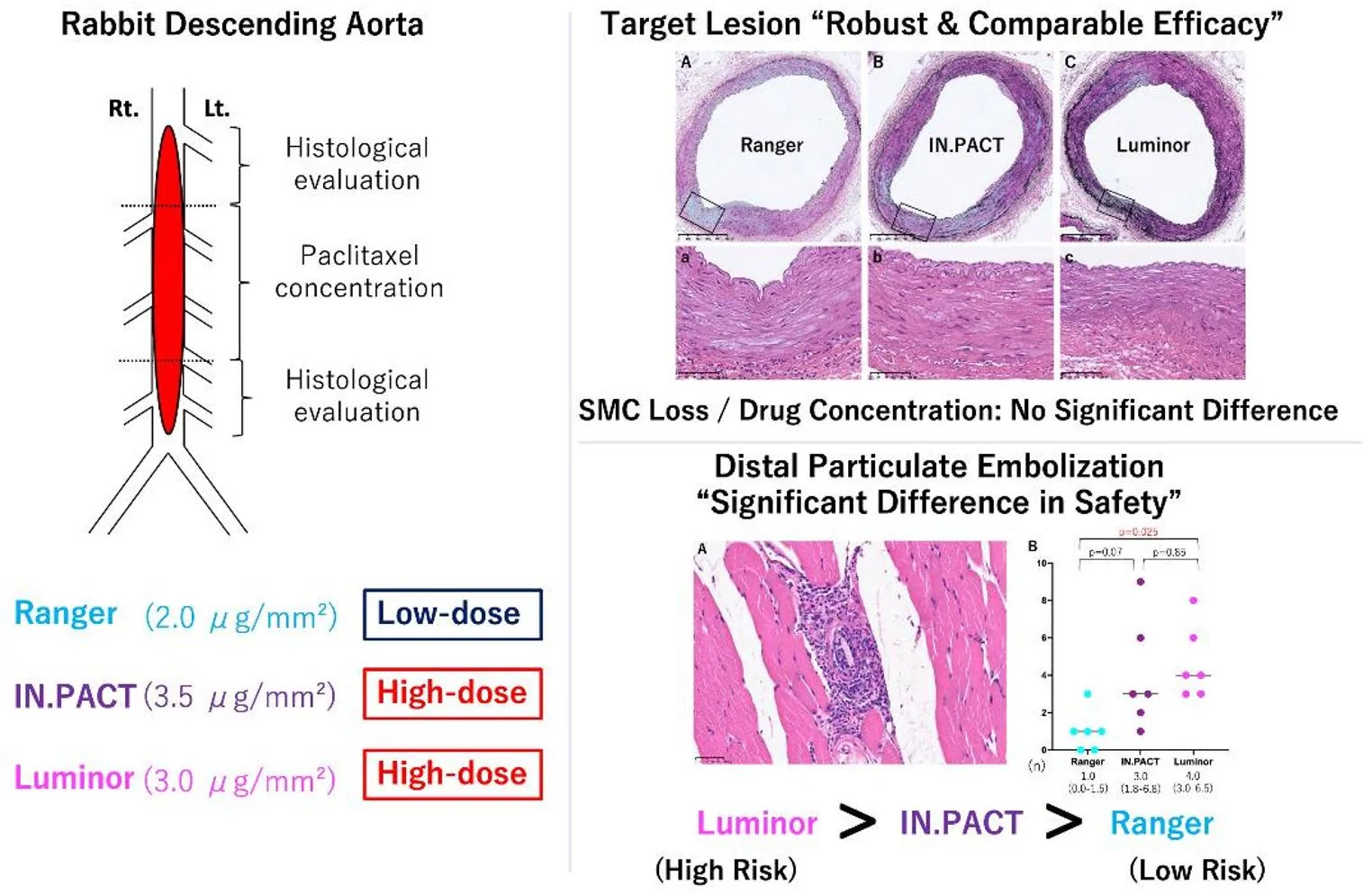

**Supplementary Information:**

The online version contains supplementary material available at https://doi.org/10.1007/s12928-026-01290-2.

## Introduction

 Endovascular treatment (EVT) for femoropopliteal (FP) arteries have become a cornerstone in the treatment of peripheral artery disease (PAD) [1]. Drug-coated balloons (DCBs) have emerged as a transformative technology, demonstrating significantly improved primary patency and reduced target lesion revascularization (TLR) compared to balloon angioplasty (BA) in multiple randomized controlled trials [2–4]. While drug-coated balloons (DCBs) have significantly improved patency rates in the femoropopliteal artery, concerns have been raised regarding the impact of distal particulate embolization on clinical outcomes. Specifically, in patients with chronic limb-threatening ischemia (CLTI), there is an ongoing discussion that embolic particles carrying high-dose paclitaxel may impede microcirculatory perfusion and wound healing, potentially correlating with observations of increased minor amputation rates in certain clinical settings [5, 6]. However, the exact biological contribution of these particles to clinical limb events remains to be fully elucidated, necessitating a detailed histological evaluation of different DCB coating technologies.

Currently, there are no large-scale, head-to-head randomized clinical trials directly comparing the contemporary DCBs available on the market, which differ significantly in paclitaxel dose and excipient technologies. Prominent devices include the low-dose Ranger (2.0 µg/mm², citrate ester excipient), the high-dose IN.PACT Admiral (3.5 µg/mm², urea excipient), and the Luminor (3.0 µg/mm², ester excipient). The current study aimed to histologically and biologically compare the local biologic drug effect and the degree of distal particulate embolization among these three distinct DCBs using a standardized healthy rabbit descending aorta model, while adhering to their respective recommended inflation protocols.

## Methods

### Rabbit model of descending aorta balloon angioplasty

**Fig. 1:**
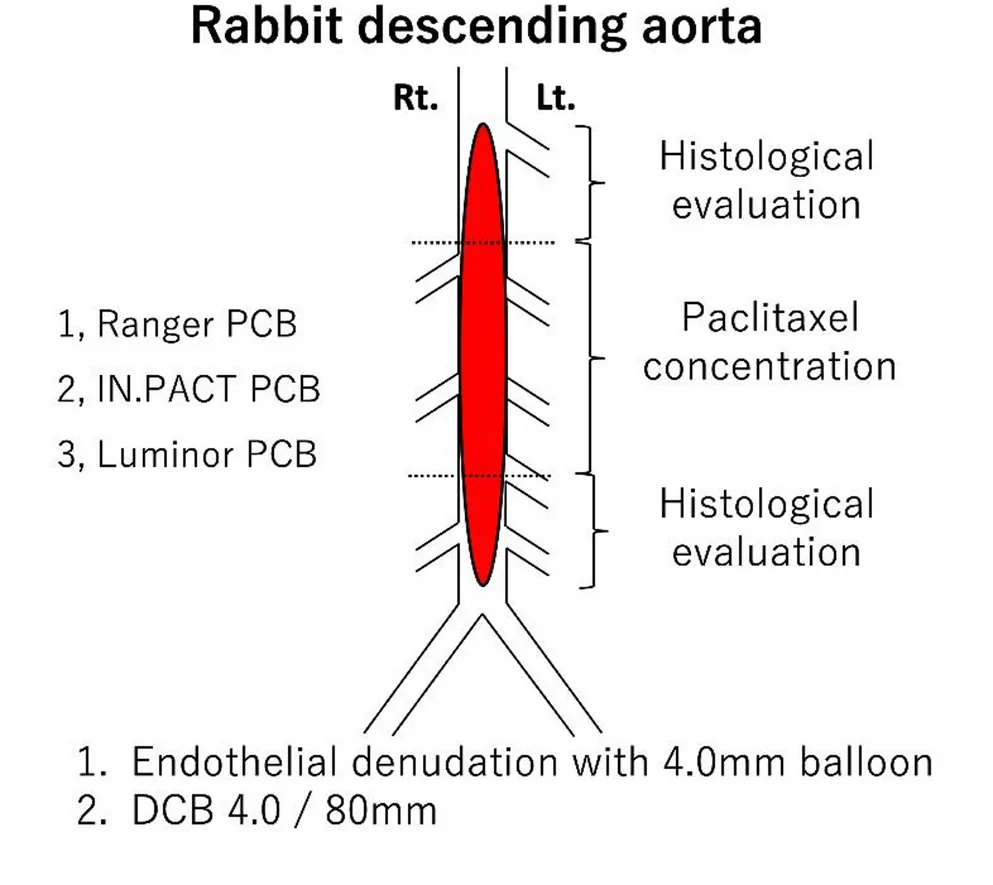
Study protocol. Schematic diagram. The treated descending aorta was separated into three parts 28 days following DCB use: the proximal and distal part for histopathological evaluations and the mid part for measurements of paclitaxel concentrations. Rt., right; Lt., left; DCB, drug-coated balloon

The study protocol was reviewed and approved by the institutional Animal Care and Use Committee (reference number: 250156). Eighteen Japanese white rabbits were fed a normal diet and received dual-antiplatelet therapy (aspirin and clopidogrel) starting 2 days prior to the procedure and continued until the day of sacrifice. General anesthesia was maintained with isoflurane via an inhalation mask, and surgical access was obtained via the right carotid artery using a sterile technique to insert a 5 F vascular sheath. Following systemic administration of nitroglycerin and heparin, baseline angiography of the descending aorta was performed. Endothelial denudation was induced using a 4.0 × 20 mm compliant balloon catheter inflated at nominal pressure, which was slipped from the distal descending artery to promote neointimal proliferation (Fig. 1).

Subsequently, a 4.0 × 80 mm DCB was advanced to the target lesion. The balloon was inflated at a target balloon-to-artery overstretch ratio of 1:1.1 to 1.2. A total of 18 rabbits were equally divided into three groups: the Ranger group (*n* = 6), the IN.PACT group (*n* = 6), and the Luminor group (*n* = 6). To reflect clinical instructions for use (IFU), the Ranger and IN.PACT DCBs were inflated for 180 s, whereas the Luminor DCB was inflated for 60 s. Following DCB deflation, completion angiography was performed to document vessel patency (Table 1).

**Table 1:** Morphometric analysis of the treated arteries

Parameter	Ranger	IN.PACT	Luminor	*R* vs. L	*R* vs. I	I vs. L
				*p*-value		
EEL area, mm^2^	4.0 (3.6–5.2)	4.0 (3.6–5.2)	3.7 (3.4–4.2)	0.99	0.73	0.69
IEL area, mm^2^	2.5 (2.1–3.2)	2.7 (2.4–3.7)	2.5 (2.2–3.9)	0.97	0.87	0.73
lumen area, mm^2^	2.5 (2.1–3.1)	2.5 (2.3-3.0)	2.4 (1.9–2.9)	0.93	0.99	0.93
medial area, mm^2^	1.1 (1.0-1.2)	1.2 (1.2–1.3)	1.2 (1.2–1.2)	0.33	0.067	0.61
intimal area, mm^2^	0.09 (0.03–0.15)	0.25 (0.11–0.56)	0.10 (0.05–0.26)	0.88	0.078	0.18
% stenosis, %	3.3 (1.4–5.5)	10.6 (5.5–16.2)	2.9 (1.8–10.5)	0.80	0.07	0.22

EEL, external elastic lamina; IEL, internal elastic lamina; R, Ranger; I, IN.PACT; L, Luminor

### Collection of rabbit descending aorta and distal skeletal muscles

**Fig. 2:**
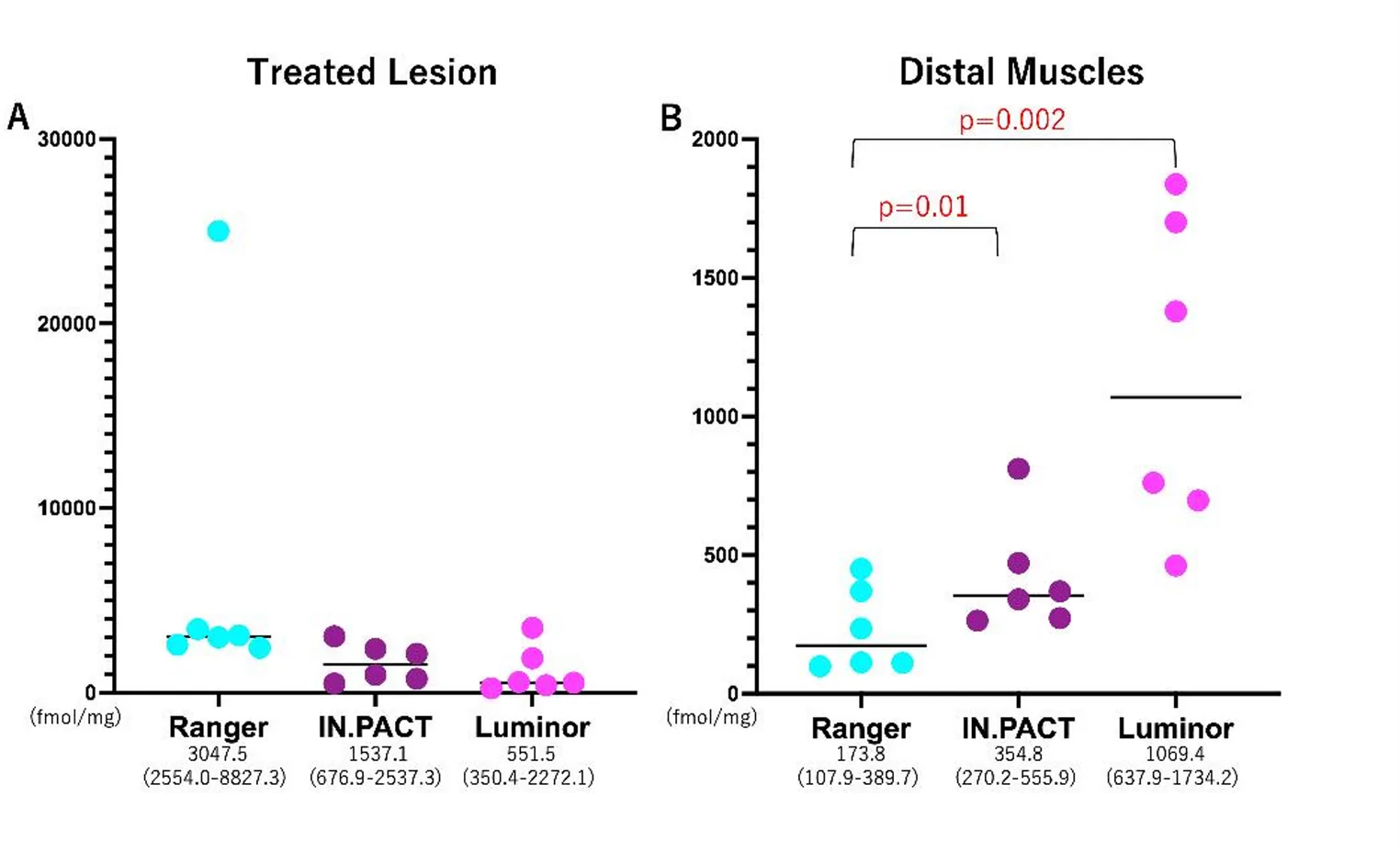
Paclitaxel concentrations of treated lesions and distal skeletal muscles 28 days after drug-coated balloon use. (**A**) In the treated lesion of the distal aorta, paclitaxel concentrations were the highest in the Ranger group, followed by the IN.PACT and Luminor groups, although there were no significant differences. (**B**) In skeletal muscles distal of the treated vessel, paclitaxel concentrations were the highest in the Luminor group, followed by the IN.PACT and Ranger groups

Twenty-eight days following the index procedure, the animals were anesthetized, and follow-up angiography was performed to confirm patency. The treated descending aorta was flushed with 1 L of lactated Ringer’s solution. The target lesion was then identified using angiographic landmarks and divided into three sections: both proximal and distal parts for histopathological evaluations and a mid part for the measurement of paclitaxel concentrations. Downstream skeletal muscle samples were harvested from the bilateral regions below the knee, specifically targeting the tibialis anterior and peroneal muscles. Each muscle was similarly divided into two parts for parallel histological evaluation and bioanalysis (Fig. 2).

### Histology and histomorphometry

**Fig. 3:**
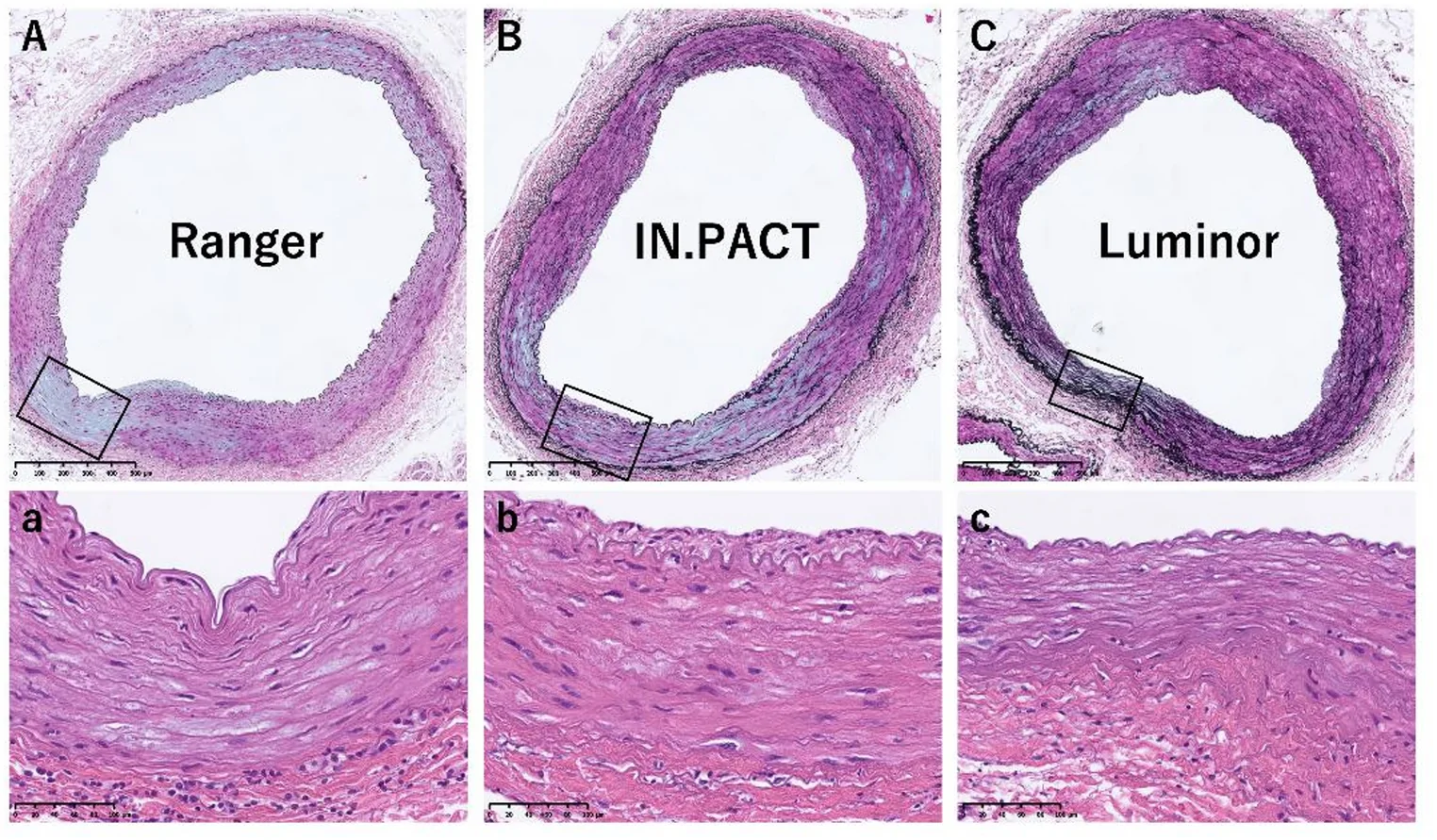
Representative pictures of descending aortas 28 days after drug-coated balloon use. Low-power (upper row, A to C, Movat Pentachrome stain) and high-power (lower row, a to c, hematoxylin and eosin stain) images of descending aortas

For histopathological analysis, the proximal and distal parts of the treated descending aorta and the skeletal muscle samples were fixed with 10% formalin perfusion, subdivided, and embedded in paraffin. Histological sections of 4 to 6 μm thickness were mounted on charged slides and stained with hematoxylin and eosin, as well as Movat Pentachrome as previously performed [7–13]. Biologic drug effects were assessed using a semi-quantitative medial smooth muscle cell (SMC) loss scoring system (range 0–4) assessing both depth and circumference. Furthermore, the circumferential angle of severe SMC loss—defined as the angle of medial SMC loss exceeding 50% of the medial depth—was measured using computer-assisted software. The downstream skeletal muscles were examined for signs of vasculitis and inflammatory cell reaction to assess distal particulate embolization. All histomorphometric analyses were performed by an independent observer blinded to the treatment allocations (Fig. 3) (Table 2).

**Table 2:** Pathological analysis of the treated arteries and distal skeletal muscles

Parameter	Ranger	IN.PACT	Luminor	*R* vs. L	*R* vs. I	I vs. L
				*p*-value		
SMC loss score (depth)	3.8 (2.3-4.0)	3.2 (2.3–3.8)	2.7 (2.3–3.5)	0.477	0.789	0.856
SMC loss score (circumference)	2.7 (2.0-3.5)	2.3 (2.2–2.8)	2.0 (1.7–2.4)	0.110	0.490	0.588
angle of severe SMC loss (degree)	60.3 (4.5–128.2)	85.5 (7.8–128.4)	45.4 (27.3–93.7)	0.188	0.656	0.610
fibrin/platelet thrombus score	0.0 (0.0–0.0)	0.0 (0.0–0.0)	0.0 (0.0–0.0)	NA		
neointimal fibrin score	0.0 (0.0–0.0)	0.0 (0.0–0.0)	0.0 (0.0–0.0)	NA		
medial proteoglycan score	3.0 (1.6–3.7)	1.5 (1.3–1.8)	1.7 (1.3–1.8)	0.027	0.021	0.988
calcification score	0.00 (0.00–0.25)	0.00 (0.00–1.42)	0.00 (0.00–0.42)	0.650	0.952	0.823
inflammation score	1.3 (1.2–1.8)	1.7 (1.3–1.8)	1.5 (1.3–1.8)	0.899	0.899	1.000
hemorrhage score	0.0 (0.0–0.0)	0.0 (0.0–0.0)	0.0 (0.0–0.0)	NA		
medial injury score	0.0 (0.0–0.0)	0.0 (0.0–0.0)	0.0 (0.0–0.0)	NA		
no. of arteries with distal changes	1.0 (0.0–1.5)	3.0 (1.8–6.8)	4.0 (3.0–6.5)	0.025	0.072	0.851

SMC, smooth muscle cell; R, Ranger; I, IN.PACT; L, Luminor; NA, not applicable

### Bioanalysis of paclitaxel concentrations

The mid parts of the treated aorta and the corresponding downstream skeletal muscle samples were kept frozen at -80 °C until bioanalysis. The treated aorta and downstream skeletal muscles were weighed and subsequently homogenized in 1 mL acetonitrile using a Shake Master Neo (Bio Medical Science, Tokyo, Japan). Homogenized samples were centrifuged to collect supernatants. The supernatants were then dried using a SpeedVac concentrator (Thermo Fischer Scientific, Waltham, MA, USA) and reconstituted in 100 µL of 75% acetonitrile in ultrapure water. A 2-µL volume of the 10-fold diluted sample was injected into liquid chromatography-tandem mass spectrometry (LC-MS) (Table 3).

**Table 3:** Paclitaxel concentraion of the treated arteries and distal skeletal muscles

Paclitaxel concentration (fmol/mg)	Ranger	IN.PACT	Luminor	*R* vs. L	*R* vs. I	I vs. L
				*p*-value		
At target lesion	3047.5 (2554.0-8827.3)	1537.1 (676.9-2537.3)	551.5 (350.4-2272.1)	0.213	0.266	0.989
At distal skeletal muscles	173.8 (107.9-389.7)	354.8 (270.2-555.9)	1069.4 (637.9-1734.2)	0.0016	0.010	0.640

R, Ranger; I, IN.PACT; L, Luminor

The quantitative analysis of paclitaxel concentrations was performed using an LCMS-8050 triple quadrupole mass spectrometer coupled to a UHPLC-Nexera system (Shimadzu, Kyoto, Japan). Samples were separated using a 2.0 × 150 mm, 1.9 μm Hypersil (Waltham, MA, United States of America) Gold C18 column (Thermo Fischer Scientific). Mobile phase A consisted of ultrapure water containing 0.1% acetic acid and 0.05% formic acid, and mobile phase B was 95% acetonitrile. Isocratic elution with 50% mobile phase B at a flow rate of 0.3 mL/min was employed. Mass spectrometric detection of paclitaxel was conducted in positive electrospray ionization mode. Other mass spectrometric parameters were as follows: flow rate of nebulizer gas, 3.0 L/min; flow rate of heating gas, 10.0 L/min; flow rate of drying gas, 10.0 L/min; interface temperature, 300 °C; and heat block temperature, 400 °C.

### Statistical analysis

Continuous data are presented as the median with interquartile range (IQR). The normality of distributions was evaluated using the Shapiro-Wilk test. Comparisons of variables with nonparametric distributions among the three groups were performed using the Kruskal-Wallis test, followed by the Steel-Dwass post hoc analysis for multiple comparisons. A p-value of < 0.05 was considered statistically significant. All statistical analyses were conducted using appropriate analytical software.

## Results

### Biologic drug effect in the target lesion

Quantitative analysis of paclitaxel concentrations in the treated descending aortas demonstrated active drug transfer across all devices. Although the median paclitaxel concentration in the target lesion appeared highest in the Ranger group [3047.5 fmol/mg (interquartile range: 2554.0–8827.3)], followed by the IN.PACT [1537.1 fmol/mg (676.9–2537.3)] and Luminor [551.5 fmol/mg (350.4–2272.1)] groups, there was no statistically significant difference among the three groups.

**Fig. 4:**
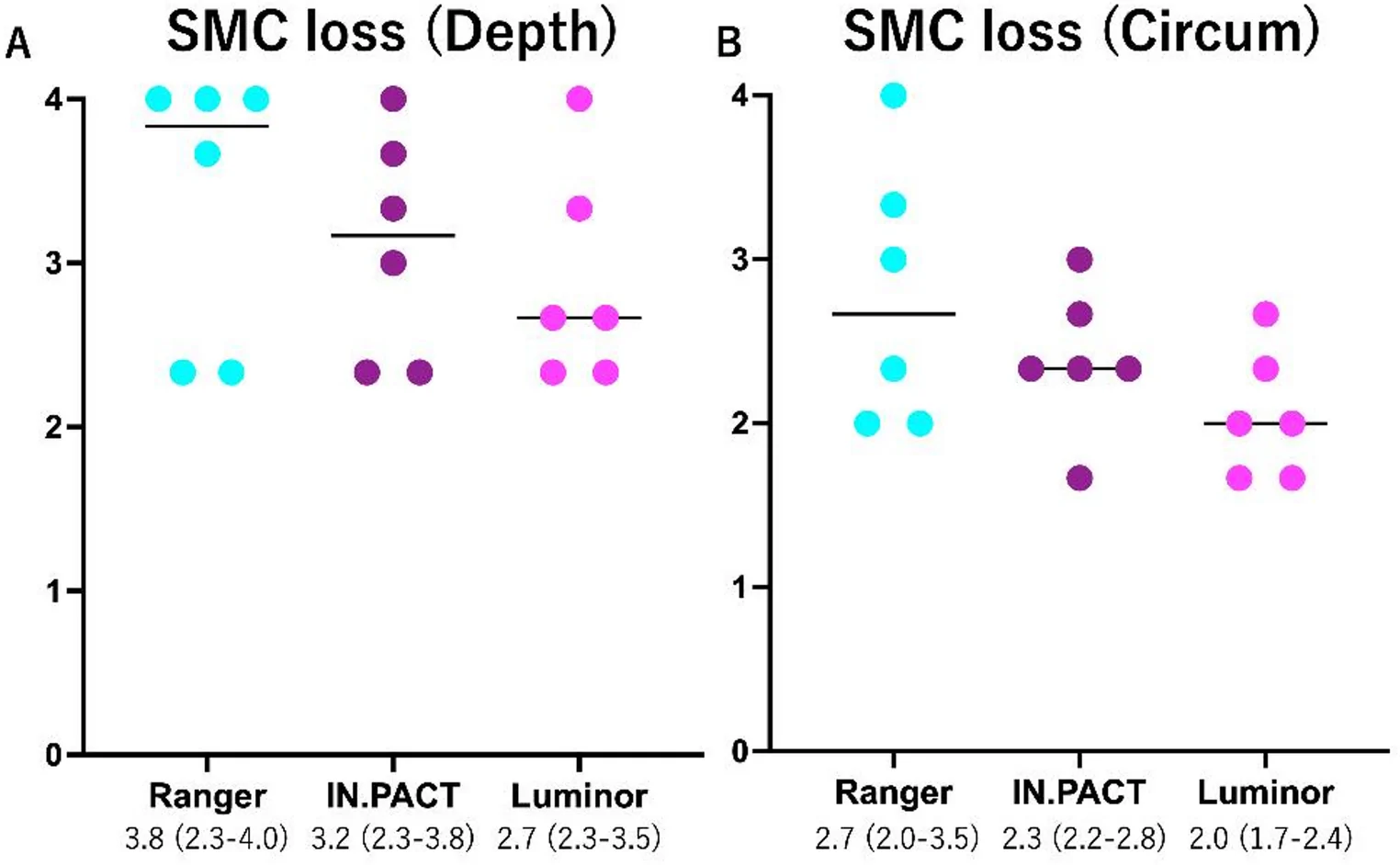
Histological scores of SMC loss (depth and circumference). (**A**) The score for the depth of medial SMC loss was the highest in the Ranger group, followed by the IN.PACT and Luminor groups. (**B**) The score for the circumference of medial SMC loss was the highest in the Ranger group, followed by the IN.PACT and Luminor groups. SMC, smooth muscle cell

Consistent with the pharmacokinetic findings, histopathological evaluations demonstrated robust biological effects without significant differences across the devices. The semi-quantitative score for medial smooth muscle cell (SMC) loss depth was 3.8 (2.3–4.0) for Ranger, 3.2 (2.3–3.8) for IN.PACT, and 2.7 (2.3–3.5) for Luminor. Quantitative analysis of the circumferential angle of severe medial SMC loss showed a median of 85.5° (7.8–128.4°) in the IN.PACT group, 60.3° (4.5–128.2°) in the Ranger group, and 45.4° (27.3–93.7°) in the Luminor group, with no statistically significant differences observed among the groups. Furthermore, the medial proteoglycan score was significantly higher in the Ranger group [3.0 (1.6–3.7)] compared to the IN.PACT [1.5 (1.3–1.8), *p* = 0.021] and Luminor [1.7 (1.3–1.8), *p* = 0.027] groups. Other histomorphometric safety parameters in the target lesion showed no significant differences, and findings such as fibrin/platelet thrombus or hemorrhage were scarcely observed across all groups (Fig. 4).

### Evaluation of distal particulate embolization

In stark contrast to the comparable efficacy in the target lesions, bioanalysis of the downstream distal skeletal muscles revealed drastic differences in distal drug washout. The paclitaxel concentration was overwhelmingly highest in the Luminor group [1069.4 fmol/mg (637.9–1734.2)], followed by the IN.PACT [354.8 fmol/mg (270.2–555.9)] and Ranger [173.8 fmol/mg (107.9–389.7)] groups.

**Fig. 5:**
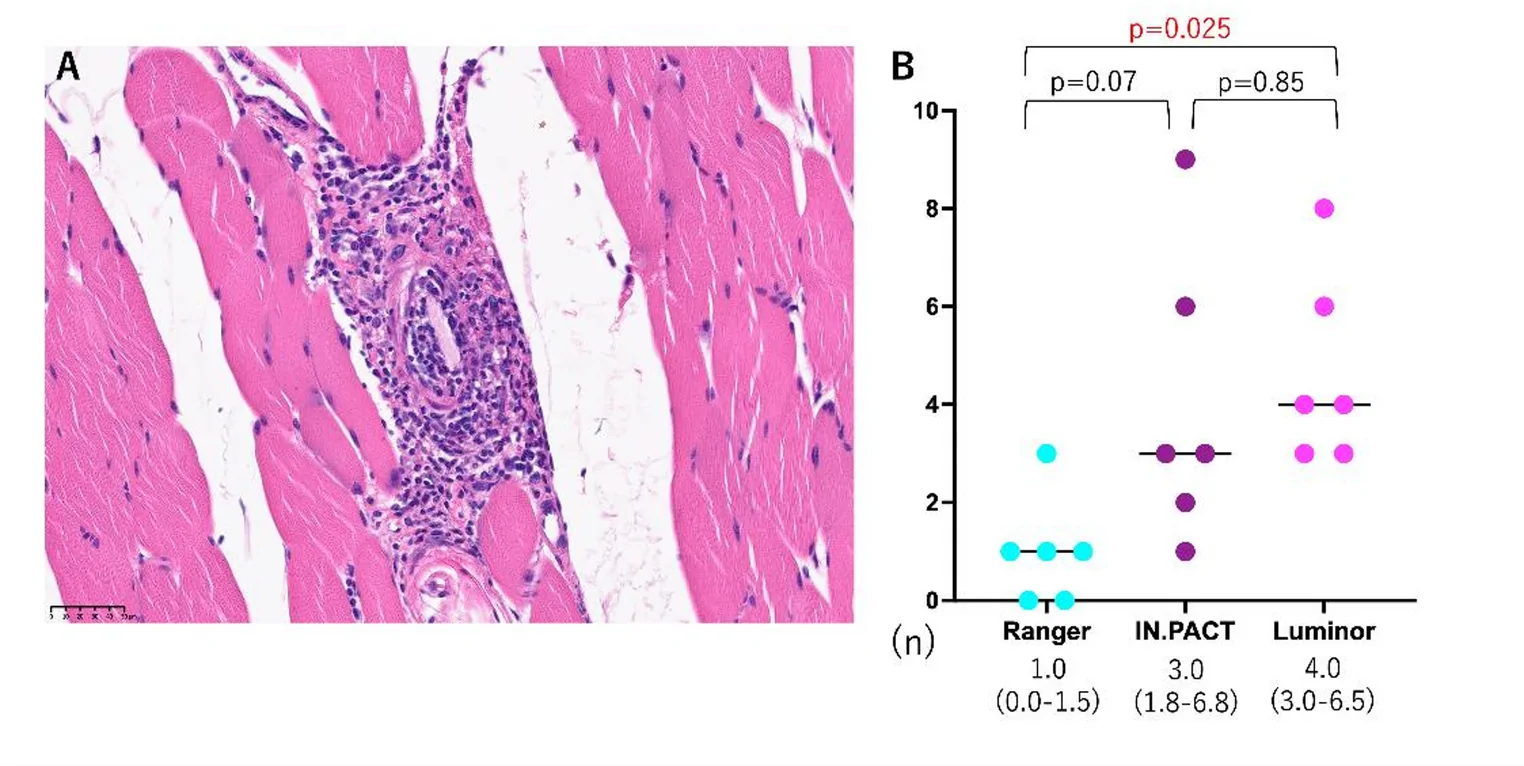
Vasculitis due to distal particulate emboli after Luminor use and number of arteries with distal changes. Number of arteries with distal changes was significantly less in Ranger compared with IN.PACT and Luminor

Histological examination of the downstream muscles correlated directly with these pharmacokinetic results. The incidence of distal particulate embolization, evaluated by the number of arteries with distal changes, was notably severe in the Luminor group [median 4.0 (3.0–6.5)]. This incidence of distal embolization was significantly higher compared to the Ranger group [1.0 (0.0–1.5)] (*p* = 0.025). The IN.PACT group demonstrated an intermediate frequency of histological distal changes [3.0 (1.8–6.8)] (Fig. 5).

## Discussion

The current study histologically and biologically compared three contemporary DCBs with distinct coating technologies and drug dosages. The principal findings are twofold: first, all three devices—Ranger, IN.PACT, and Luminor—exhibited comparable local biologic drug effects and target lesion pharmacokinetics without statistically significant differences. Second, and in stark contrast to the local efficacy, the degree of distal particulate embolization differed significantly among the devices. The low-dose Ranger DCB maintained a significantly lower embolic burden, whereas the high-dose IN.PACT and Luminor DCBs were associated with a higher risk of downstream embolization.

Regarding local efficacy, quantitative analysis revealed that the low-dose Ranger DCB (2.0 µg/mm²) achieved local paclitaxel tissue concentrations and histological smooth muscle cell loss equivalent to the high-dose IN.PACT (3.5 µg/mm²) and Luminor (3.0 µg/mm²) DCBs. Additionally, our histological analysis revealed a significantly higher medial proteoglycan score in the Ranger group. Proteoglycan accumulation in the media is considered one of the surrogate markers of paclitaxel efficacy, reflecting drug-induced extracellular matrix alterations. These findings underscore that the absolute dose of paclitaxel is not the sole determinant of biologic efficacy. All tested devices successfully delivered therapeutic levels of paclitaxel to the vessel wall, inducing robust inhibition of neointimal hyperplasia consistent with the established class effect of DCBs demonstrated in previous clinical trials compared to plain old balloon angioplasty [3, 4, 14]. Notably, the comparable efficacy between the low-dose Ranger and high-dose IN.PACT observed in our preclinical model mirrors the results of recent real-world clinical cohort studies, such as the PROSPECT MONSTER study [15]. This alignment between preclinical data and clinical outcomes not only corroborates the non-inferiority of the low-dose platform but also reaffirms the translational value of the healthy rabbit model in predicting the relative clinical performance of contemporary DCBs. This suggests that efficient excipient technology, such as the citrate ester coating in Ranger, facilitates effective drug transfer even at lower doses.

While local efficacy was comparable, safety profiles regarding distal particulate embolization varied significantly. A striking observation was the higher degree of histologically confirmed embolic vasculitis and skeletal muscle paclitaxel concentrations in the groups treated with high-dose DCBs (Luminor and IN.PACT). A higher drug load may lead to a greater amount of drug being washed downstream. However, drug dose alone does not fully explain our findings, because distal embolization tended to be more pronounced with Luminor than with IN.PACT, although this difference did not reach statistical significance, despite both being high-dose DCBs. Our results suggest that additional device-specific factors, particularly coating integrity and formulations, may also play an important role in determining the extent of distal particulate embolization. In addition, the shorter 1-minute inflation time recommended in the Luminor IFU may have influenced the initial drug-transfer profile and may have contributed to loosely adherent drug particles washing downstream [16, 17]. In contrast, the highly stable excipient formulation of the Ranger DCB appears to effectively minimize downstream embolic washout. These findings highlight the critical importance of drug load, coating integrity, and procedural conditions in contributing to distal particulate embolization.

The clinical implications of distal particulate embolization are particularly significant in the treatment of below-the-knee (BTK) arteries. Our previous pathological analysis of BTK lesions revealed a high prevalence of medial arterial calcification and a unique occlusion mechanism characterized by organized thrombus and embolic debris, leading to chronic total occlusion (CTO) [18–21]. Given that the BTK vascular bed is already compromised by these pathological features, the additional “emboli” of drug-coated particles from DCBs may further jeopardize microcirculatory perfusion and impede wound healing in patients with CLTI [5, 6]. While recent large-scale data have led to a more neutral perspective on the association between paclitaxel and late mortality [22, 23], the risk of minor or major amputations remains a critical concern. Our study demonstrates that the extent of distal embolic load and the subsequent inflammatory response differ significantly among DCB coatings. Therefore, selecting a DCB platform that minimizes distal debris may be a crucial strategy to preserve the already vulnerable distal outflow and improve limb-salvage outcomes in clinical practice.

### Limitations

This study has several limitations. First, it utilized a healthy rabbit descending aorta model, which does not fully replicate the pharmacokinetics of drug transfer in highly calcified human atherosclerotic lesions. In highly calcified lesions, the dense calcium acts as a physical barrier that limits drug penetration, while complex morphologies like calcified nodules or organized thrombus may cause uneven balloon apposition, potentially increasing the risk of coating flaking and particulate shedding. Although our healthy animal model lacks these complex features, this controlled environment ensures a uniform baseline across all devices, enabling a more direct and unbiased comparison of the drug effect and embolization risks of each DCB. Second, the difference in IFU-based inflation times (1 min versus 3 min) may act as a procedural confounder in the direct comparison of the coatings. Nevertheless, the observation that such differences in embolic burden occurred when strictly adhering to clinical IFUs highlights the critical need to consider both coating integrity and procedural variables to ensure safety in real-world endovascular practice.

## Conclusion

In conclusion, our study demonstrated significant differences in distal particulate embolization and subsequent vascular responses among different DCB coating technologies. Given that the BTK vascular bed is pathologically characterized by a high burden of medial calcification and a propensity for embolic occlusion, the additional embolic load from certain DCB platforms may further compromise distal outflow. These findings provide mechanistic insights and may have implications for device selection in patients with compromised distal runoff; however, clinical studies are needed to confirm their impact on perfusion and limb outcomes.

## Electronic Supplementary Material

Below is the link to the electronic supplementary material.

Supplementary Material 1
