# Supplementary material for: Comparative analysis of biologic drug effects and distal particulate embolization among three drug-coated balloons: a histological and biological study in a rabbit model
Source: Cardiovasc Interv Ther. 2026 Jun 9;41(4):1068–76. doi: 10.1007/s12928-026-01290-2 (PMC13582060; doi:10.1007/s12928-026-01290-2)
Supplement: Supplementary file 1 [file 12928_2026_1290_MOESM1_ESM.docx]

**Supplemental Table 1. Characteristics of each DCBs**

|  | Ranger | IN.PACT | Luminor |
| --- | --- | --- | --- |
| Company | Boston Scientific | Medtronic | iVascular |
| Drug | PTX | PTX | PTX |
| Drug dose (μg/mm²) | 2.0 | 3.5 | 3.0 |
| Excipient | ATBC | Urea | Water Reduced Ester |
| PTX formulation | Micro-crystalline | Crystalline | Crystaline |
| Balloon state during Drug deposition | Pulverisation | Micro-pipetting | Spray |
| Technique of Drug deposition | Deflated | Inflated | Not specified |
